# A COMPREHENSIVE EVIDENCE-BASED INTERVENTION PROGRAMME SIGNIFICANTLY REDUCES INTENSIVE CARE UNIT-ACQUIRED WEAKNESS AND IMPROVES FUNCTIONAL RECOVERY: A RETROSPECTIVE ANALYSIS

**DOI:** 10.2340/jrm.v57.43563

**Published:** 2025-10-26

**Authors:** Hongrui ZHU, Yueming ZHANG, YAN ZHOU, Hongxia YAN

**Affiliations:** 1 The Fourth Affiliated Hospital of Qiqihar Medical College, Harbin; 2 Department of Critical Care Medicine, The Second Affiliated Hospital of Qiqihar Medical College, Qiqihar City, Heilongjiang Province, PR China

**Keywords:** ICU-acquired weakness, evidence-based medicine, ICU, before-and-after study

## Abstract

**Background:**

Intensive care unit-acquired weakness (ICU-AW) affects 25–50% of critically ill patients, resulting in prolonged hospitalization and impaired functional recovery. Despite recognition of its clinical importance, effective prevention and treatment strategies remain limited.

**Objective:**

To evaluate the efficacy of a comprehensive evidence-based intervention programme on ICU-AW incidence and functional outcomes compared with standard care.

**Methods:**

This retrospective analysis conducted between May 2021 and December 2023 included 420 critically ill patients allocated to either an evidence-based intervention group (*n* = 200) receiving a structured programme incorporating early mobilization, respiratory rehabilitation, swallowing training, psychological support, and nutritional optimization, or a standard care group (*n* = 220). Primary outcomes included ICU-AW incidence and hospital length of stay. Secondary outcomes comprised MRC muscle strength scores, nutritional status (Subjective Global Assessment), and functional independence (Barthel Index).

**Results:**

The evidence-based intervention group demonstrated significantly lower ICU-AW incidence (32.5% vs 57.3%, *p* < 0.001) and shorter hospital stays (18.4±6.2 days vs 25.7±7.8 days, *p* < 0.001) compared with standard care. Post-intervention MRC scores were significantly higher in the evidence-based intervention group (50.4±5.9 vs 44.2±7.1, *p* < 0.001), representing a mean improvement of 7.3 points (95% CI: 6.2–8.4) compared with 1.9 points (95% CI: 1.2–2.6) in standard care. Nutritional status improved by at least one SGA grade in 56% of evidence-based intervention patients vs 28.6% of standard care patients (*p* < 0.001). Barthel Index scores increased substantially more in the evidence-based intervention group (32.3-point increase vs 13.4-point increase, *p* < 0.001), with 41% achieving scores > 75 compared with 16% in standard care (*p* < 0.001).

**Conclusion:**

A comprehensive evidence-based intervention programme significantly reduces ICU-AW incidence and improves muscle strength, nutritional status, and functional outcomes in critically ill patients. This multimodal strategy offers promise for alleviating ICU-AW’s burden and warrants broader clinical adoption.

## LAY ABSTRACT

When patients spend time in intensive care units, they often develop muscle weakness that makes it hard to move and care for themselves. This condition, called intensive care unit-acquired weakness, affects about 1 in 3 critically ill patients. In this study, we tested whether a comprehensive care program could help prevent and treat this weakness. The program included early physical exercises, breathing training, mental health support, and better nutrition planning. We compared 200 patients who received this special program with 220 patients who received standard intensive care unit care. Our results showed that patients in the special program were much less likely to develop severe weakness (32.5% versus 57.3%). They also got stronger faster, stayed in the hospital for shorter periods, and were better able to care for themselves when they left. This research suggests that a well-planned, multi-faceted approach to intensive care unit care can significantly improve patients' recovery and quality of life.

Intensive care unit-acquired weakness (ICU-AW) is a prevalent and debilitating complication that affects critically ill patients, particularly those requiring mechanical ventilation and advanced life support systems. This condition is closely associated with sophisticated medical interventions, including extracorporeal membrane oxygenation (ECMO) and continuous renal replacement therapy (CRRT) (1, 2). ICU-AW manifests as a spectrum of symptoms including muscle atrophy, quadriplegia, decreased reflexes, and difficulty in weaning from mechanical ventilation, which can severely impact patient outcomes and overall well-being (3).

The pathophysiology of ICU-AW is complex, involving rapid alterations in skeletal muscle proteostasis that result in substantial muscle deterioration and weakness. This process is exacerbated by the prolonged immobility and systemic inflammation often experienced by critically ill patients. Such interplay necessitates sophisticated, multifaceted interventions (4).

Recent research has identified several effective interventions for both preventing and treating ICU-AW. A comprehensive review and meta-analysis by Anekwe et al. demonstrated that initiating rehabilitation early in the treatment process substantially lowers the occurrence of ICU-AW (5). Furthermore, a randomized standard trial conducted by García-Pérez-de-Sevilla et al. indicated that neuromuscular electrical stimulation can shorten hospitalization times for patients with ICU-AW while enhancing muscle strength (6).

Despite the positive effects of nutrition and exercise on patients with ICU-AW, most existing studies tend to focus on isolated aspects of treatment, lacking a comprehensive approach to intervention. This underscores the necessity for further exploration into a holistic nursing plan tailored for patients suffering from ICU-AW. Evidence-based medicine (EBM) provides a framework for integrating the most reliable clinical data with individual clinical expertise and patient values to deliver the highest quality of care. The application of evidence-based intervention programmes has proven beneficial in managing critically ill patients within ICUs, yielding positive clinical outcomes such as reducing infection rates and enhancing nursing satisfaction (7).

Given the significant impact of ICU-AW on patient outcomes and the potential benefits of evidence-based interventions, there is a critical need for research examining the effects of comprehensive, evidence-based intervention protocols on patients experiencing ICU-AW. Accordingly, this investigation evaluates an EBI programme’s impact on muscle strength, nutritional status, self-care abilities, and quality of life.

## AIMS AND OBJECTIVES OF THE STUDY

The primary aim of this retrospective analysis was to evaluate the impact of a previously implemented EBI programme on patients with severe ICU-acquired weakness (ICU-AW). Specifically, the study sought to:

1. Assess the effectiveness of the EBI programme in reducing the prevalence of ICU-AW among critically ill patients.
2. Examine the influence of the EBI programme on hospital stay duration and swallowing function in ICU patients.
3. Evaluate improvements in key clinical outcomes, including:
  - Muscle strength (Medical Research Council [MRC] score);
  - Nutritional status (subjective global assessment [SGA]);
  - Self-care abilities (Barthel Index [BI]).
4. Compare the outcomes of patients who received the EBI programme with those who received standard ICU care during the study period.

The overarching objective was to determine whether the implementation of evidence-based interventions as part of a quality improvement initiative could enhance rehabilitation outcomes and improve the quality of life for ICU patients at risk of or suffering from ICU-AW. This retrospective analysis aimed to provide evidence supporting the potential clinical benefits of EBI programmes as a standard component of ICU care, paving the way for future prospective studies and broader clinical adoption.

## DESIGN AND METHOD

### Study design and subjects

This retrospective, single-institution analysis was conducted between May 2021 and December 2023, adhering to ethical principles outlined in the 1964 Declaration of Helsinki and its subsequent amendments. The study received approval from the Institutional Review Board of the Fourth Affiliated Hospital of Qiqihar Medical College Ethics Committee (approval number: 2024KYPJ065). Informed consent was waived due to the retrospective nature of the research, which involved anonymized clinical data without direct patient intervention or biological specimen collection.

The study was designed as a retrospective analysis of a quality improvement initiative implemented as part of standard clinical practice. This approach allowed for evaluation of real-world implementation outcomes while minimizing researcher influence on clinical decisions. The study population consisted of 420 critically ill patients systematically allocated into 2 groups: the EBI Group (*n* = 200) who received a structured evidence-based intervention programme, and the Standard care group (*n* = 220) who received standard ICU care before the EBI programme was fully implemented.

### Setting and sample

The study was conducted in the intensive care units of the Fourth Affiliated Hospital of Qiqihar Medical College, a tertiary care teaching hospital with a 26-bed medical-surgical ICU. Sample size determination was based on previously published studies examining ICU-AW incidence as the primary outcome. Assuming an ICU-AW incidence of 60% in the standard care group and anticipating a reduction to 40% in the intervention group, with α = 0.05 and β = 0.20 (power = 80%), a minimum of 190 patients per group was required. The enrolment target was increased to 200 patients per group to account for potential data loss or protocol violations.

The study employed specific inclusion and exclusion criteria to ensure a well-defined patient cohort:

Inclusion criteria:

- Adults aged 18 years or older.
- First-time ICU admission with an anticipated stay exceeding 72 h.
- Cognitively alert patients capable of following at least 3 commands (e.g., eye-opening/closing, tongue protrusion, head nodding).
- Haemodynamically stable.

Exclusion criteria:

- Conditions contraindicating assessment, such as unstable fractures or significant physical disabilities.
- Pre-existing neuromuscular disorders (e.g., Guillain-Barré syndrome, myasthenia gravis) or intracranial/spinal pathologies affecting motor function.
- Pregnancy, advanced malignancy, active haemorrhage, or poor prognosis.
- Recent acute myocardial infarction or severe craniocerebral injuries.
- Conditions precluding functional exercise participation (e.g., cervical spine or limb fractures).

Patient selection followed a consecutive sampling approach to minimize selection bias. All patients meeting the inclusion criteria during the study period were considered for enrolment.

### Intervention methods

The intervention group underwent a structured EBI programme developed through evidence-based medicine methodologies. The programme was formulated through a systematic review of literature published between 2015 and 2021 in PubMed, EMBASE, and Cochrane databases, using search terms including “ICU-acquired weakness”, “critical illness myopathy”, “early mobilization”, and “nutritional support”. The evidence synthesis was conducted by a multidisciplinary team including critical care physicians, rehabilitation specialists, nutritionists, and ICU nurses, resulting in an integrated protocol ranked by strength of evidence.

The program consisted of 5 key components:

1. *Physical assessment and functional exercises:* Patients underwent standardized strength assessments within 48 h of meeting eligibility criteria, followed by early mobilization facilitated by trained staff. This progressive mobility programme included passive range of motion exercises advancing to active-assisted and active exercises as tolerated, with a targeted duration of 20–30 min twice daily.
2. *Pulmonary rehabilitation training:* Structured respiratory therapy was undertaken, including guided exercises and expectoration techniques under nursing supervision. Protocols incorporated inspiratory muscle training using threshold devices calibrated to 30% of maximal inspiratory pressure, diaphragmatic breathing exercises, and bronchial drainage techniques based on individual patient needs.
3. *Psychological support:* Meditation sessions took place with soothing music for relaxation, conducted daily for 15–20 min using standardized audio recordings delivered via headphones. Content was selected based on evidence for reducing anxiety and promoting relaxation in critical care settings (e.g., guided imagery, mindfulness meditation, and nature sounds).
4. *Extubation strategy:* Personalized plans were followed, based on muscle strength and cough intensity to minimize extubation failure rates. This component involved systematic assessment of MRC scores and cough peak flow measurements prior to weaning attempts. Patients with MRC scores > 48 and adequate cough strength (peak flow > 60 L/min) were considered for extubation following standard weaning protocols.
5. *Swallowing function training:* Ice stimulation exercises were given before meals to improve swallowing ability. The protocol included thermal-tactile stimulation with ice, followed by progressive trials with varying fluid consistencies under speech therapy supervision. Patients were monitored for signs of aspiration using validated bedside swallowing assessments.

Additionally, personalized nutritional recovery strategies were developed for each patient following the European Society of Clinical Nutrition and Metabolism (ESPEN) guidelines. Nutritional interventions included protein supplementation (targeting 1.5–2.0 g/kg/day), individualized caloric goals based on indirect calorimetry where available or predictive equations, and micronutrient supplementation as indicated by laboratory values.

The standard care group received conventional ICU management consisting of passive range-of-motion exercises twice daily for 20 min, systematic repositioning every 2 h to prevent pressure injuries, respiratory support techniques such as chest physiotherapy and airway clearance procedures, and continuous nasogastric nutritional support. This approach represented the typical care delivered prior to the implementation of the evidence-based protocol.

Protocol adherence was monitored using a standardized checklist completed by the care team for each intervention component. Overall protocol adherence exceeded 85% for all components in the EBI group, with daily documentation in the electronic medical record. The intervention was delivered daily during the ICU stay, with a maximum duration of 2 weeks for patients with extended admissions; for those discharged earlier, protocols continued until discharge.

To implement the EBI programme, additional resources were allocated, including 1 full-time equivalent rehabilitation therapist, 0.5 full-time equivalent nutritionist for consultations, and 0.3 full-time equivalent psychologist for sessions. Multidisciplinary coordination was achieved through daily team rounds involving critical care physicians, nurses, therapists, nutritionists, and psychologists, supplemented by electronic checklists and shared digital platforms for real-time updates on patient progress, ensuring efficient integration into existing workflows without substantial disruption. This collaborative framework facilitated prompt adjustments to individual patient needs and minimized silos in care delivery.

### Data collection tools and methods

Two independent researchers extracted the study data from patient electronic medical records housed in the hospital’s digital repository. Inter-rater reliability was assessed on a 10% random sample of records, with Cohen’s kappa exceeding 0.90 for all outcome measures, indicating excellent agreement between data extractors. Discrepancies were resolved through consensus discussion with a third researcher when necessary.

Baseline demographic and clinical data included age, sex, body mass index, primary ICU admission diagnosis, comorbidities, APACHE II scores, SOFA scores, duration of mechanical ventilation, and sedation requirements. These variables were selected based on their established relevance to ICU-AW development in previous literature.

The following validated tools were used to measure key outcomes:

*MRC Strength Score:* Evaluated muscle strength across 6 muscle groups (e.g., wrist extension, knee extension). Scores ranged from 0 (no contraction) to 5 (normal strength), with a total score below 48 indicating ICU-AW. This assessment was performed by trained physiotherapists blinded to study groups. The MRC scale has demonstrated excellent inter-rater reliability (ICC = 0.94) and validity for ICU populations in previous validation studies(8).

*SGA Nutrition Assessment:* Assessed nutritional status using 3 categories: A (well-nourished), B (moderate malnutrition), and C (severe malnutrition). The SGA was conducted by certified dietitians with experience in critical care nutrition. This tool incorporates multiple parameters including weight change, dietary intake, gastrointestinal symptoms, functional capacity, and physical examination findings to generate a comprehensive nutritional profile (9).

*Barthel Index (BI):* Measured independence in daily activities. Scores ranged from 0 (completely dependent) to 100 (completely independent). The BI assessment was performed by occupational therapists trained in standardized administration procedures. This instrument evaluates 20 activities of daily living including feeding, bathing, grooming, dressing, bowel control, bladder control, toilet use, transfers, mobility, and stairs(10).

*Swallowing function test:* Assessed swallowing ability using a standardized water swallow test involving observation for choking or aspiration. The test was administered by speech-language pathologists following a standardized protocol. Patients were observed while swallowing 3 mL, 5 mL, 10 mL, and 20 mL of water, with assessment for coughing, voice quality changes, and oxygen desaturation.

### Observation targets

The study evaluated multiple outcome measures categorized as primary and secondary endpoints. Primary outcomes included the incidence of ICU-acquired weakness (ICU-AW) as determined by MRC scores below 48, and the duration of hospitalization calculated from ICU admission to hospital discharge. Secondary outcomes comprised muscle strength quantified using the MRC score, nutritional status assessed via SGA classification, self-care ability measured by the Barthel Index, and swallowing function evaluated through standardized water swallow testing. All outcomes were assessed at baseline (within 48 h of meeting eligibility criteria) and post-intervention (on ICU discharge for ICU-specific measures or hospital discharge for length of stay and functional outcomes, with a maximum observation window of 2 weeks aligned with the intervention delivery period).

### Data analysis

*Statistical analysis.* Statistical analyses were performed using IBM SPSS Statistics for Windows, version 26.0 (IBM Corp, Armonk, NY, USA). The normality of continuous data distribution was assessed using the Shapiro–Wilk test and visual inspection of histograms and Q–Q plots. Continuous variables with normal distribution are presented as mean± standard deviation (SD), while non-normally distributed variables are expressed as median with interquartile range (IQR). Categorical variables are presented as frequencies and percentages.

Sample size determination was based on previously published studies examining ICU-AW incidence as the primary outcome. Assuming an ICU-AW incidence of 60% in the standard care group and anticipating a reduction to 40% in the intervention group, with α = 0.05 and β = 0.20 (power = 80%), a minimum of 190 patients per group was required. The enrolment target was increased to 200 patients per group to account for potential data loss or protocol violations.

For between-group comparisons, independent *t*-tests were utilized for normally distributed continuous variables, while the Mann–Whitney *U* test was employed for non-normally distributed continuous variables. Categorical variables were compared using Pearson’s χ^2^ test or Fisher’s exact test when expected cell counts were less than 5. For within-group comparisons between baseline and post-intervention measurements, paired *t*-tests or Wilcoxon signed-rank tests were used as appropriate based on data distribution.

To control for potential confounding factors, multivariable logistic regression analysis was performed for dichotomous outcomes (e.g., ICU-AW incidence), adjusting for clinically relevant covariates including age, APACHE II score, and duration of mechanical ventilation. Results are reported as adjusted odds ratios (OR) with 95% confidence intervals (CI). For continuous outcomes, multiple linear regression models were constructed with similar adjustment variables, and results are presented as adjusted mean differences with 95% CI.

Treatment effect sizes were calculated to quantify the magnitude of intervention effects. For continuous outcomes, Cohen’s *d* was computed (small effect: 0.2–0.5, medium effect: 0.5–0.8, large effect: > 0.8). For dichotomous outcomes, risk ratios and number needed to treat (NNT) were calculated with corresponding 95% confidence intervals.

Missing data were minimal (< 5% for all primary outcomes) and were handled using multiple imputation with chained equations, creating 20 imputed datasets. Sensitivity analyses comparing complete-case analysis with results from imputed datasets revealed no substantial differences. The level of statistical significance was set at *p* < 0.05 for all analyses, and all tests were 2-sided. Results for secondary outcomes should be interpreted as exploratory when considering the overall study conclusions.

Subgroup analyses were prespecified for patients with prolonged mechanical ventilation (> 7 days), high APACHE II scores (> 25), and advanced age (> 70 years) to assess whether intervention effects were consistent across these clinically relevant subpopulations. Interaction tests were performed to evaluate whether treatment effects differed significantly between subgroups, with *p* < 0.10 considered indicative of potential effect modification.

This retrospective study was approved by the Institutional Review Board of the Fourth Affiliated Hospital of Qiqihar Medical College Ethics Committee (approval number: 2024KYPJ065). The approval ensured that the study adhered to established ethical guidelines and standards.

The research was conducted in accordance with the ethical principles outlined in the 1964 Declaration of Helsinki and its subsequent amendments. These principles emphasize respect for human dignity, beneficence, and justice in research involving human subjects.

The retrospective nature of the study involved the analysis of anonymized clinical data, which did not include direct patient intervention or biological specimen collection. As such, the Ethics Committee granted a waiver of informed consent for this research. However, written informed consent had been obtained from participants at the time of their treatment, which covered the use of their anonymized data for future research purposes.

These ethical considerations ensured that the study maintained high standards of integrity while protecting patient rights and confidentiality.

## RESULTS

### General information on patients

A total of 462 patients were initially screened for eligibility, with 42 excluded based on predefined criteria. The final analysis included 420 patients, with 200 in the EBI group and 220 in the standard care group. Baseline characteristics for both groups are presented in Table I.

**Table I:** Baseline demographic and clinical characteristics of study participants

Characteristic	EBI group (*n* = 200)	Standard care group (*n* = 220)	*p*-value
Age, years, mean ± SD	63.4 ± 12.7	64.8 ± 11.9	0.245^a^
Sex, n (%)			0.876^b^
Male	115 (57.5)	128 (58.2)	
Female	85 (42.5)	92 (41.8)	
BMI, kg/m², mean ± SD	25.2 ± 4.6	24.9 ± 4.3	0.503^a^
APACHE II score, mean ± SD	19.6 ± 5.4	20.1 ± 5.2	0.327^a^
SOFA score on admission, mean ± SD]	7.3 ± 2.8	7.5 ± 3.0	0.476^a^
Primary ICU admission diagnosis, *n* (%)			0.912^b^
Sepsis	49 (24.5)	54 (24.5)	
Respiratory failure	42 (21.0)	47 (21.4)	
Postoperative monitoring	37 (18.5)	41 (18.6)	
Cardiovascular disorders	31 (15.5)	36 (16.4)	
Neurological disorders	24 (12.0)	25 (11.4)	
Other	17 (8.5)	17 (7.7)	
Mechanical ventilation, *n* (%)	146 (73.0)	165 (75.0)	0.642^b^
Duration of mechanical ventilation prior to enrolment, days, median (IQR)	5.4 (3.2–8.7)	5.7 (3.0–9.2)	0.568^c^
Vasopressor use, *n* (%)	112 (56.0)	129 (58.6)	0.580^b^
Baseline MRC score, mean ± SD	43.1 ± 6.2	42.3 ± 6.7	0.218^a^

a Independent *t*-test.

b Pearson's χ² test or Fisher's exact test.

c Mann-Whitney *U* test.

The mean age of participants was 63.4± 12.7 years in the EBI group and 64.8± 11.9 years in the standard care group. Gender distribution was comparable between groups, with 57.5% male participants in the EBI group and 58.2% in the standard care group. The most common admission diagnoses were sepsis (24.5%), respiratory failure (21.2%), and postoperative monitoring (18.6%), with similar distribution between groups. Baseline APACHE II scores, indicating illness severity, were 19.6± 5.4 and 20.1± 5.2 in the EBI and standard care groups, respectively.

Statistical analysis revealed no significant differences between the groups (*p* > 0.05) for any baseline characteristics.

This initial balance is crucial for attributing any subsequent differences in outcomes to the interventions rather than pre-existing group disparities, there-by enhancing the validity of the study’s findings. All enrolled patients completed the study protocol, with no withdrawals or lost follow-up data. As this retrospective analysis utilized comprehensive electronic medical records, all patients were followed until hospital discharge or death on an intention-to-treat basis, with no study-related withdrawals. For the 44 in-hospital deaths (18 in EBI, 26 in standard care), last-observation-carried-forward was applied for incomplete assessments. No inter-institutional transfers occurred, ensuring complete data capture.

### Incidence of ICU-AW, length of hospital stays, and swallowing dysfunction

Following the intervention, significant differences in key outcomes were observed between the two groups, as presented in Table II. The EBI group demonstrated a markedly reduced length of hospital stay compared with the standard care group (mean 18.4± 6.2 days vs 25.7± 7.8 days, *p* < 0.001). This represents a reduction of 7.3 days (95% CI: 5.9–8.7) in total hospitalization time, a clinically significant finding with implications for resource utilization and healthcare costs. Similarly, ICU length of stay was significantly shorter in the EBI group (9.2±4.1 days vs 13.5±5.6 days, *p* < 0.001).

**Table II:** Primary outcomes comparison between EBI and standard care group

Outcome	EBI group (*n* = 200)	Standard care group (*n* = 220)	Mean difference/Risk ratio (95% CI)	*p*-value
Hospital length of stay, days, mean ± SD	18.4 ± 6.2	25.7 ± 7.8	–7.3 (–8.7 to –5.9)	< 0.001^a^
ICU length of stay, days, mean ± SD]	9.2 ± 4.1	13.5 ± 5.6	–4.3 (–5.2 to –3.4)	< 0.001^a^
Incidence of ICU-AW, *n* (%)	65 (32.5)	126 (57.3)	0.57 (0.45 to 0.71) *	< 0.001^b^
Adjusted OR for ICU-AW (95% CI)†	[Added: Reference]	2.79 (1.86 to 4.18)	—	< 0.001
Swallowing dysfunction, *n* (%)	37 (18.5)	82 (37.3)	0.50 (0.35 to 0.70)*	< 0.001^b^
Mechanical ventilation duration, days, median (IQR)	6.8 (4.2–10.3)	9.6 (5.8–14.2)	–2.8 (–3.9 to –1.7)‡	< 0.001^c^
Reintubation rate, *n* (%)	14 (7.0)	31 (14.1)	0.50 (0.27 to 0.90)*	0.018^b^
In-hospital mortality, *n* (%)	18 (9.0)	26 (11.8)	0.76 (0.43 to 1.35)*	0.349^b^

a Independent *t*-test.

b Pearson's χ² test or Fisher's exact test.

c Mann-Whitney *U* test.

Moreover, the incidence of ICU-AW was significantly lower in the EBI group (32.5% vs 57.3%, *p* < 0.001). When adjusted for baseline characteristics including age, APACHE II score, and duration of mechanical ventilation, the odds ratio for developing ICU-AW in the standard care group compared with the EBI group was 2.79 (95% CI: 1.86–4.18), further supporting the protective effect of the intervention.

Additionally, swallowing function was notably improved in the EBI group relative to the standard care group, with swallowing dysfunction observed in 18.5% of patients in the EBI group compared with 37.3% in the standard care group (*p* < 0.001). Subgroup analysis revealed that the greatest benefit in swallowing function was observed in patients who had undergone mechanical ventilation for more than 7 days (reduction in dysfunction rate from 52.8% to 26.4%, *p* < 0.001), suggesting particular efficacy of the swallowing training protocol in this high-risk population.

The mechanical ventilation duration was also significantly shorter in the EBI group (median 6.8 days, IQR 4.2–10.3) compared with the standard care group (median 9.6 days, IQR 5.8–14.2), with a median difference of 2.8 days (95% CI: 1.7–3.9, *p* < 0.001). Furthermore, the reintubation rate was reduced by 50% in the EBI group (7.0% versus 14.1%, *p* = 0.018), though no significant difference was observed in in-hospital mortality (9.0% vs 11.8%, *p* = 0.349). These results affirm the EBI programme’s efficacy in ameliorating critical illness complications.

### MRC muscle strength scores of the 2 groups

MRC scores were evaluated for both groups before and after the intervention, with results displayed in Table III. Initially, no statistically significant difference was observed in MRC scores between the standard and EBI groups (42.3±6.7 vs 43.1±6.2, *p* = 0.218). Baseline scores indicated that approximately 58% of all enrolled patients met the criteria for ICU-AW (MRC < 48) at study entry, consistent with previously reported prevalence rates in similar critical care populations.

**Table III:** MRC muscle strength scores before and after intervention

Time point	EBI group (*n* = 200)	Standard care group (*n* = 220)	Between-group *p*-value
Baseline, mean ± SD	43.1 ± 6.2	42.3 ± 6.7	0.218^a^
Post-intervention, mean ± SD	50.4 ± 5.9	44.2 ± 7.1	< 0.001^a^
Mean change (95% CI)	7.3 (6.2–8.4)	1.9 (1.2–2.6)	< 0.001^a^
Within-group *p*-value	< 0.001^b^	< 0.001^b^	
Percentage of patients with MRC ≥ 48, *n* (%)			
Baseline	83 (41.5)	86 (39.1)	0.613^c^
Post-intervention	135 (67.5)	94 (42.7)	< 0.001^c^

a Independent *t*-test.

b Pearson's χ² test or Fisher's exact test.

c Mann-Whitney *U* test.

Post-intervention analysis, however, revealed significantly higher MRC scores in the EBI group compared with the standard care group (50.4± 5.9 vs 44.2± 7.1, *p* < 0.001). This represents a mean improvement of 7.3 points (95% CI: 6.2–8.4) in the EBI group vs only 1.9 points (95% CI: 1.2–2.6) in the standard care group. The difference in improvement was statistically significant (*p* < 0.001), with a large effect size (Cohen’s *d* = 0.94).

Furthermore, the proportion of patients with MRC scores ≥ 48 (indicating absence of ICU-AW) increased from 41.5% to 67.5% in the EBI group, compared with a modest increase from 39.1% to 42.7% in the standard care group (*p* < 0.001 for between-group comparison). When analysed by muscle group, improvements were most pronounced in the upper limbs (mean increase of 1.3 points per muscle group) compared with the lower limbs (mean increase of 1.1 points per muscle group) in the EBI group, potentially reflecting the greater challenge in mobilizing lower extremities in critically ill patients. These findings underscore the EBI’s efficacy in bolstering muscle strength.

### SGA nutritional assessment of the 2 groups

Prior to the intervention, there was no significant difference in the SGA grade distribution between the 2 groups (*p* = 0.347), as detailed in Table IV. At baseline, 42% of patients in the EBI group and 45% in the standard care group were classified as moderately malnourished (Grade B), while 28% and 30%, respectively, were classified as severely malnourished (Grade C), reflecting the high prevalence of nutritional compromise in critical illness.

**Table IV:** SGA nutritional status before and after intervention

SGA Grade	EBI group (*n* = 200)		Standard care group (*n* = 220)		*p*-value
	Baseline, *n* (%)	Post-intervention, *n* (%)	Baseline, *n* (%)	Post-intervention, *n* (%)	
**A** (Well-nourished)	60 (30.0)	116 (58.0)	55 (25.0)	70 (31.8)	0.347^a^ (baseline)
**B** (Moderately malnourished)	84 (42.0)	64 (32.0)	99 (45.0)	97 (44.1)	< 0.001^a^ (post)
**C** (Severely malnourished)	56 (28.0)	20 (10.0)	66 (30.0)	53 (24.1)	
Improvement by ≥1 grade, *n* (%)	—	112 (56.0)	—	63 (28.6)	< 0.001^b^

a Independent *t*-test.

b Pearson's χ² test or Fisher's exact test.

However, following the intervention, the EBI group exhibited a notable improvement in SGA grade compared with the standard care group, with a statistically significant difference (*p* < 0.001). Post-intervention, the proportion of well-nourished patients (Grade A) increased from 30% to 58% in the EBI group, compared with an increase from 25% to 32% in the standard care group. Similarly, the proportion of severely malnourished patients (Grade C) decreased from 28% to 10% in the EBI group, vs a smaller reduction from 30% to 24% in the standard care group.

Overall, 56% of patients in the EBI group showed improvement by at least one SGA grade, compared with only 28.6% in the standard care group (*p* < 0.001). Multivariate analysis identified baseline albumin levels and early initiation of enteral nutrition (within 24 h of eligibility) as independent predictors of nutritional improvement (adjusted OR 1.86, 95% CI: 1.32–2.61, *p* < 0.001), suggesting these factors as potential targets for future nutritional interventions. This highlights the pivotal role of targeted nutritional support in critical care.

### BI scores for both groups

Before the intervention, statistical analysis indicated no significant difference in BI scores between the 2 groups (32.4± 12.6 vs 31.8± 13.1, *p* = 0.643), as outlined in Table V. Baseline functional status was similarly compromised in both groups, with over 75% of patients requiring moderate to maximum assistance for basic activities of daily living, reflecting the substantial functional impairment associated with critical illness.

**Table V:** BI scores before and after intervention

BI assessment	EBI group (*n* = 200)	Standard care group (*n* = 220)	Between-group *p*-value
Baseline score, mean ± SD	32.4 ± 12.6	31.8 ± 13.1	0.643^a^
Post-intervention score, mean ± SD	64.7 ± 14.3	45.2 ± 15.1	< 0.001^a^
Mean change (95% CI)	32.3 (29.8–34.8)	13.4 (11.2–15.6)	< 0.001^a^
Within-group *p*-value	< 0.001^b^	< 0.001^b^	
Functional independence category, *n* (%)			
Baseline			0.782^c^
Severe dependence (BI 0–40)	126 (63.0)	142 (64.5)	
Moderate dependence (BI 41–60)	56 (28.0)	62 (28.2)	
Mild dependence (BI 61–99)	18 (9.0)	16 (7.3)	
Independence (BI 100)	0 (0.0)	0 (0.0)	
Post-intervention			< 0.001^c^
Severe dependence (BI 0–40)	32 (16.0)	98 (44.5)	
Moderate dependence (BI 41–60)	86 (43.0)	87 (39.5)	
Mild dependence (BI 61–99)	82 (41.0)	35 (15.9)	
Independence (BI 100)]	0 (0.0)	0 (0.0)	

a Independent *t*-test.

b Pearson's χ² test or Fisher's exact test.

c Mann-Whitney *U* test.

However, after the intervention, the EBI group achieved significantly higher BI scores than the standard group (64.7±14.3 vs 45.2±15.1, *p* < 0.001). This represents a mean improvement of 32.3 points (95% CI: 29.8–34.8) in the EBI group compared with 13.4 points (95% CI: 11.2–15.6) in the standard care group, with the between-group difference being highly significant (*p* < 0.001) and demonstrating a large effect size (Cohen’s *d* = 1.34). The distribution of functional independence categories showed significant differences post-intervention (*p* < 0.001). While the majority of patients in both groups presented with severe dependence at baseline (63.0% in EBI group, 64.5% in standard care group), post-intervention only 16.0% of EBI patients remained severely dependent compared with 44.5% of standard care patients. Notably, 41.0% of patients in the EBI group achieved mild dependence status (BI 61–99) compared with only 15.9% in the standard care group.

Correlation analysis revealed a significant positive association between improved MRC scores and enhanced BI scores (*r* = 0.72, *p* < 0.001), suggesting that increases in muscle strength directly contribute to improved functional outcomes and underscoring the importance of strength training in rehabilitation protocols.

Importantly, by discharge, 41% of patients in the EBI group achieved BI scores > 75, indicating minimal assistance requirements for activities of daily living, compared with only 16% in the standard care group (*p* < 0.001). Such gains hold profound implications for post-discharge autonomy.

## DISCUSSION

ICU-AW is a neuromuscular complication that can prolong the period of mechanical ventilation in patients, elevate mortality rates, and impose a substantial financial burden on patients’ families (11). Therefore, enhancing interventions for ICU-AW is crucial. How-ever, the pathophysiological mechanism underlying the ICU-AW is completely unclear, contributing to a lack of effective preventive measures, which is also the main reason for the high incidence of ICU-AW (12). In prior studies, several factors have been identified as contributing to the development of ICU-AW. These include prolonged mechanical ventilation, administration of vasoactive agents, sedative use, nutritional deficiencies, and insufficient rehabilitation efforts (13, 14). However, existing studies have yielded varying conclusions and recommendations due to their diverse focal points. Although clinical efforts to address the factors contributing to ICU-AW have yielded some positive results, the overall outcomes remain suboptimal, possibly due to a narrow focus on specific parameters rather than a comprehensive approach. At present, there is no clinical intervention programme for ICU-AW. Therefore, this current study attempts to establish a complete intervention programme grounded in EBM, hoping that the EBI programme can bring more significant benefits to patients. The EBI programme provided in this study includes 3 key aspects: early rehabilitation, psychological intervention, and early nutrition support.

The findings of this study indicate that the EBI programme significantly reduces the incidence of ICU-AW (32.5% vs 57.3%, *p* < 0.001, as shown in Table II), and shortens the duration of ICU stays (9.2± 4.1 days vs 13.5± 5.6 days, *p* < 0.001) and hospital length of stay (18.4± 6.2 days vs 25.7± 7.8 days, *p* < 0.001), representing a clinically significant 7.3-day reduction in hospitalization. The comprehensive intervention also substantially enhanced muscle strength (MRC score improvement of 7.3 points vs 1.9 points, *p* < 0.001, as demonstrated in Table III), self-care abilities (Barthel Index increase of 32.3 points vs 13.4 points, *p* < 0.001, Table V), and nutritional status (56% of patients improved by at least one SGA grade vs 28.6% in standard care, *p* < 0.001, Table IV). These results underscore the critical importance of implementing the EBI programme.

Early rehabilitation is considered critically important in ICU inpatients (15). Prior research has established a significant correlation between ICU-AW and both functional outcomes and quality of life for patients. For example, a study by Eggmann et al. (16) showed significant differences in functional outcomes and hospital length of stay among patients’ frailty levels, as assessed by the 6-minute walk (*p* = 0.013; distances of 110 m, 196 m, and 222.5 m) and the Functional Independence Measure (*p* = 0.001; scores of 91, 113, and 112). These findings emphasize the importance of focusing on functional recovery in ICU patients. Therefore, the EBI protocol implemented in this study emphasizes the early recovery of patients. The medical team provided patients with early pulmonary rehabilitation and progressive mobility protocols, starting with passive range of motion and advancing to active exercises as tolerated. This approach resulted in significant improvement in MRC scores, with 67.5% of patients in the EBI group achieving scores ≥ 48 post-intervention compared with only 42.7% in the standard care group (*p* < 0.001, Table III). Moreover, the functional independence categories improved substantially, with 41% of EBI patients achieving mild dependence status (BI 61–99) compared with only 15.9% in the standard care group (Table V).

The value of early rehabilitation was also further confirmed in a randomized standard trial conducted by Watanabe et al. (17), which demonstrated that the timing of initiating functional exercise is associated with patient outcomes; specifically, an earlier start of rehabilitation resulted in better outcomes. Therefore, this study suggests that the medical team should carefully evaluate the patient’s condition and capabilities to initiate rehabilitation training as early as possible.

Previous studies have shown that ICU patients frequently experience anxiety and depression, which can negatively impact their quality of life (17). Therefore, the EBI programme incorporated targeted psychological intervention for patients. Medical staff provided patients with structured meditation sessions using standardized audio recordings for 15–20 min daily, which contributed to the comprehensive improvement observed across multiple outcome domains. Although the limitations of this study prevented the collection of specific patients’ mental health scores, there is no doubt that a better mental state is associated with better rehabilitation outcomes. Research by Kredentser et al. supports this notion, demonstrating that psychological intervention, such as maintaining a diary, can help reduce anxiety and depression in ICU patients while improving patients with traumatic stress (18). Although there is no clear evidence for the relationship between mental health and the occurrence of ICU-AW, this study still suggests that psychological intervention has important supplementary therapeutic value for ICU patients.

Malnutrition leads to generalized muscle atrophy, reduces patient immunological resistance, and prolongs recovery periods. Therefore, the optimization of nutritional support for critically sick patients is an indispensable part of clinical work. Relevant studies have shown that the mortality rate of ICU patients with inadequate nutrition (defined as receiving less than 50% of predicted calorie requirements) during the first week is 1.7 times higher than that of patients with adequate nutrition (> 80% of predicted calorie requirements) (19). In response to this critical issue, the EBI programme implemented personalized nutritional recovery strategies following ESPEN guidelines, with protein supplementation targeting 1.5–2.0 g/kg/day and individualized caloric goals. The significant improvement in nutritional status is clearly demonstrated in Table IV, with the proportion of well-nourished patients (SGA Grade A) increasing from 30% to 58% in the EBI group compared with only 25% to 32% in the standard care group. Similarly, the proportion of severely malnourished patients (Grade C) decreased substantially from 28% to 10% in the EBI group vs a smaller reduction from 30% to 24% in the standard care group (*p* < 0.001). The value of nutritional support was further confirmed by the study of Verceles et al., who provided a protein-supported intervention for ICU patients (20). Their results showed that their intervention method not only reduced the occurrence of cognitive impairment but also promoted the nitrogen balance in patients.

Our findings also revealed a significant correlation between improved MRC muscle strength scores and enhanced Barthel Index scores (*r* = 0.72, *p* < 0.001), suggesting that increases in muscle strength directly contribute to improved functional outcomes. This relationship underscores the importance of our multimodal approach, which addresses both the physical and nutritional aspects of recovery. Additionally, the EBI group showed a 50% reduction in reintubation rates (7.0% vs 14.1%, *p* = 0.018) and significantly shorter mechanical ventilation duration (median 6.8 days vs 9.6 days, *p* < 0.001), further demonstrating the comprehensive benefits of our intervention programme.

In conclusion, this study successfully constructed an EBI protocol for ICU patients and confirmed its value in improving patient outcomes. However, several limitations must be acknowledged. First, the study was conducted at a single centre, which may restrict the generalizability of the findings due to the limited number of patients enrolled. Second, despite achieving statistical balance in baseline characteristics between groups, the retrospective design introduces potential for selection bias that cannot be completely eliminated. Additionally, the retrospective nature of this study introduces inherent methodological limitations that may affect the interpretation and extrapolation of results. Despite these limitations, the consistency and magnitude of benefits observed across multiple outcome measures, including the large effect sizes for muscle strength (Cohen’s *d* = 0.94) and functional independence (Cohen’s *d* = 1.34), provide robust evidence supporting implementation of this comprehensive protocol.

We hope that future research should focus on conducting large-scale, multi-centre prospective studies to further validate the efficacy of the EBI protocol. Such studies would provide more robust evidence for the widespread implementation of this comprehensive intervention approach and potentially optimize outcomes for critically ill patients.

### Conclusion

The EBI programme effectively diminishes ICU-AW incidence, curtails hospitalization duration, and augments muscle strength, nutritional status, and self-care capacity, meriting expanded clinical application.

### Limitations

This study, while providing valuable insights into the effectiveness of the EBI programme in reducing ICU-AW and improving patient outcomes, has certain limitations that warrant consideration. The retrospective design, though practical for analysing existing data, limits the ability to establish direct causal relationships between the intervention and observed outcomes. A prospective RCT would provide stronger evidence. Additionally, the study was conducted at a single institution, which may restrict the generalizability of findings to other settings with diverse patient populations or clinical practices. The exclusion of specific patient groups, such as those with pre-existing neuromuscular disorders or severe injuries, further narrows the applicability of the results to broader ICU populations. While short-term outcomes were comprehensively assessed, the lack of long-term follow-up data on functional recovery and quality of life leaves gaps in understanding the sustained impact of the EBI programme. Subjective tools, despite standardization, may introduce variability. Lastly, the absence of a cost-effectiveness analysis limits insights into the financial feasibility of implementing such programmes in resource-limited settings, though preliminary resource estimates suggest modest additional staffing needs. Despite these limitations, the study’s structured methodology and positive results highlight its potential for clinical application and pave the way for future research to address these gaps.

### Implications and recommendations for practice

The EBI programme demonstrated significant clinical implications for ICU practice through its comprehensive approach combining early rehabilitation, psychological support, and nutritional interventions. The programme’s success in reducing ICU-acquired weakness incidence, shortening ICU stays, and improving muscle strength and self-care abilities underscores the importance of implementing structured protocols in critical care settings. Nursing staff should prioritize early patient assessment and initiation of rehabilitation training while incorporating psychological interventions such as meditation to support mental health outcomes. The emphasis on early nutritional support through regular assessments and timely interventions proves crucial in preventing complications and reducing mortality. To strengthen these findings and facilitate widespread adoption, future research should focus on conducting large-scale, multi-centre prospective studies that can validate the EBI protocol’s efficacy across diverse healthcare settings, ultimately leading to improved care policies for critically ill patients.

## Data Availability

All data are available from the corresponding author.
